# Bacterial protein meta-interactomes predict cross-species interactions and protein function

**DOI:** 10.1186/s12859-017-1585-0

**Published:** 2017-03-16

**Authors:** J. Harry Caufield, Christopher Wimble, Semarjit Shary, Stefan Wuchty, Peter Uetz

**Affiliations:** 1 Center for the Study of Biological Complexity, Virginia Commonwealth University, Richmond, Virginia USA; 2 Department of Computer Science, University of Miami, Coral Gables, Florida USA; 3 Center for Computational Science, University of Miami, Coral Gables, Florida USA; 4 Sylvester Comprehensive Cancer Center, Miller School of Medicine, University of Miami, Miami, Florida USA

**Keywords:** Protein interactions, Interactome, Networks, Genome evolution

## Abstract

**Background:**

Protein-protein interactions (PPIs) can offer compelling evidence for protein function, especially when viewed in the context of proteome-wide interactomes. Bacteria have been popular subjects of interactome studies: more than six different bacterial species have been the subjects of comprehensive interactome studies while several more have had substantial segments of their proteomes screened for interactions. The protein interactomes of several bacterial species have been completed, including several from prominent human pathogens. The availability of interactome data has brought challenges, as these large data sets are difficult to compare across species, limiting their usefulness for broad studies of microbial genetics and evolution.

**Results:**

In this study, we use more than 52,000 unique protein-protein interactions (PPIs) across 349 different bacterial species and strains to determine their conservation across data sets and taxonomic groups. When proteins are collapsed into orthologous groups (OGs) the resulting meta-interactome still includes more than 43,000 interactions, about 14,000 of which involve proteins of unknown function. While conserved interactions provide support for protein function in their respective species data, we found only 429 PPIs (~1% of the available data) conserved in two or more species, rendering any cross-species interactome comparison immediately useful. The meta-interactome serves as a model for predicting interactions, protein functions, and even full interactome sizes for species with limited to no experimentally observed PPI, including *Bacillus subtilis* and *Salmonella enterica* which are predicted to have up to 18,000 and 31,000 PPIs, respectively.

**Conclusions:**

In the course of this work, we have assembled cross-species interactome comparisons that will allow interactomics researchers to anticipate the structures of yet-unexplored microbial interactomes and to focus on well-conserved yet uncharacterized interactors for further study. Such conserved interactions should provide evidence for important but yet-uncharacterized aspects of bacterial physiology and may provide targets for anti-microbial therapies.

**Electronic supplementary material:**

The online version of this article (doi:10.1186/s12859-017-1585-0) contains supplementary material, which is available to authorized users.

## Background

Our understanding of a protein's role in a biological system strongly depends on its placement in a network of protein-protein interactions (PPIs), or interactome. Recently, interactome data sets involving proteins from various microbial species have been constructed using experimental [1, 2] and inferred data (Table 1) while numerous databases have been created to store and disseminate this information [3–5]. Bacterial proteomes are particularly attractive subjects for interactome analysis due to their manageable size. The proteomes of many bacterial species include only a few thousand proteins, suggesting that they are about an order of magnitude smaller than their counterparts in many animals and plants. Therefore, most bacterial species provide more tractable interactomes compared to the human genome that has more than 20,000 protein coding genes [6] and more than 650,000 predicted PPIs [7].

**Table 1:** Comprehensive experimental microbial interactome sizes

Species Name	Interactome size (PPIs)	Unique OGs in proteome	Proteins in proteome	Proteins in interactome	Ref.
*C. jejuni*	11,687	1523	1623	1321	[29]
*E. coli*	2234	2563	4306	1269	[8]
*H. pylori*	3004	1280	1553	739	[12]
*M. loti*	3121	2981	7272	1804	[23]
*Synechocystis* sp.	3236	2246	3575	1920	[47]
* T. pallidum*	3649	736	1036	726	[17]
* S. cerevisiae*	4,549^a^	4794	6721	3278	[50]
* S. cerevisiae*	957^a^	4794	6721	1004	[51]
* S. cerevisiae*	1,809^a^	4794	6721	2018	[52]
* S. cerevisiae*	2,770^a^	4794	6721	1124	[53]

^a^Sambourg et al. [44] estimated the yeast interactome size to be ~37,000 PPIs, based on 3042 interactions among well-studied proteins curated from the literature

Nearly all published bacterial interactomes have been created using either the yeast two-hybrid (Y2H) system or affinity purification followed by mass spectrometry analysis (AP/MS). Although *E. coli* is the only bacterial species with a comprehensive interactome that has been studied by both Y2H [8] and AP/MS [9] methodologies a comparison of both methods surprisingly showed largely non-overlapping interaction data sets. In the Y2H data set of 2234 *E. coli* PPIs roughly 1800 were found outside of known protein complexes [8]. Similarly, roughly a third of ~1500 interactions that are thought to occur in protein complexes were detected by the Y2H approach, indicating that existing methodologies in isolation produce incomplete datasets [8].

**Fig. 1:**
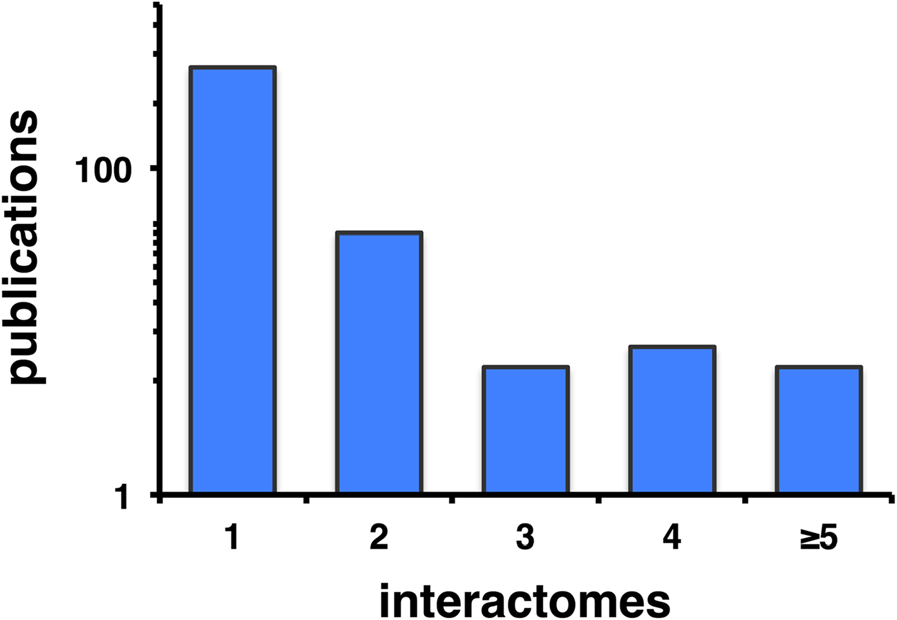
Citation analysis of the bacterial interactome literature. Publication counts include all papers that cite at least one of a total of 11 published bacterial interactome studies (as of August 2015)

A way to overcome such problems is to combine not only different datasets from the same species but also data from different species. Although cross-species interactome approaches have been recently presented for human and yeast protein sets [10] no comprehensive comparison of bacterial interactomes currently exists. While the majority of reports focus on one interactome (Fig. 1), far fewer include data from more than one set of interactions, and just two recent reports [11, 12] have investigated 6 or more out of 11 available large-scale bacterial interactome datasets. One of these studies provides an analysis of bacterial genomes in terms of their predicted functional complexity rather than the exact interactions in their interactomes [11]. Other studies dealt with four or five published interactomes (see Additional file 1 for a guide to all additional files and a complete list of interactome publications discussed here in Additional file 2), presenting only a general discussion of the evolution of protein networks [13] or a review of ways to mine high-throughput experimental data to link gene and function [14].

One of the most promising applications of interactomics is in the analysis of protein function. In a “guilt by association” approach [15, 16], PPIs provide context to proteins by considering functional roles of their known interaction partners. For example, a protein that interacts predominantly with metabolic proteins probably has a role in metabolism as well. In particular, such a method has been applied as part of the analysis of interactomics data [9, 17, 18]. As part of a guilt-by-association approach, proteins and their interaction networks may be compared through their participation in orthologous groups (OGs). Specifically, OGs are defined through non-supervised, taxonomy-limited methods [19] and reduce the complexity of interaction networks by joining proteins of similar sequence and potentially similar function. An orthology-based approach may be species-independent and can allow interaction networks of different species to be used to predict uncharacterized, conserved interactions as well as to provide an evolutionary basis for the reasons an interaction may not be present. Although analyses of conserved networks have been performed [20], in some cases alongside interactome studies [21], they have generally been limited by low proteome coverage in the underlying interactomes.

Here, we combine experimentally-derived, previously published PPIs from 349 bacterial species and strains to form a consensus meta-interactome, using orthologous groups (OG) of proteins to combine all known interactions into a single network. Notably, we observe that such a network shares characteristics of single species interactomes. Furthermore, the augmentation of single species interaction networks with a bacterial meta-interactome boosts our ability to predict functions of the underlying proteins, given its dramatically increased information content. Finally, we utilize such a bacterial meta-interactome to predict interactome sizes of species for which incomplete interaction data is available.

## Results

### The bacterial meta-interactome resembles individual interactomes in structure

**Fig. 2:**
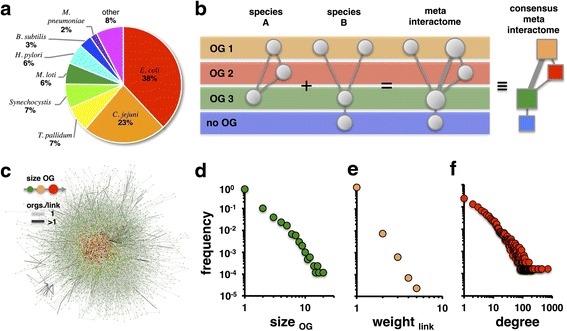
Consensus meta-interactome. **a** A breakdown of source species of the meta-interactome shows that PPIs in *E. coli* or *C. jejuni* contributed to more than half of the total set of interactions in the meta-interactome. **b** We defined the pool of interactions between proteins in different bacteria as the meta-interactome. To account for homologous proteins, we considered groups of orthologous proteins (OG) as nodes in a consensus meta-interactome. In particular, we weighted links between OGs by the underlying number of observed interactions between proteins in such groups. **c** The main component of the consensus meta-interactome pools 88.9% of all OGs. Our graphical depiction suggests that the majority of OGs consist of one protein, while such groups are mostly linked by one underlying PPI. **d** More quantitatively, we found that the majority of OGs in the consensus meta-interactome indeed have only one protein while a minority of groups includes many proteins. **e** The distributions of the number of PPI that connect proteins in different OGs (**f**) as well as the number of neighboring OGs decay as a power-laws

To compare interactions across multiple species, we first mapped proteins to orthologous groups (OGs; for details see Methods). As a source of information about OGs, we utilized the EggNOG database [19], expanding the idea of clusters of orthologous groups [22] constructed from numerous organisms. As a source of PPIs in bacteria we utilized the IntAct database [3]. Furthermore, we accounted for the protein interactome of *Mesorhizobium loti* [23], a PPI data set that was not available in the IntAct database. In particular, we accounted for all experimental sources of PPIs, suggesting that the majority of interactions (>60%) have been found in *E. coli* and *C. jejuni* (Fig. 2a). Based on the total set of roughly 52,000 interactions between proteins in the underlying organisms, we merged their OGs, resulting in a meta-interactome with nodes and edges of differing weights (Fig. 2b). In total, we obtained a consensus meta-interactome of 8475 orthologous groups that are embedded in web of 43,545 weighted links, covering 349 distinct bacterial species and strains (Fig. 2c, see Additional file 3 for details). Such a network consisted of 205 connected components that included 1352 self-connected nodes. Moreover, the largest component pooled 88.9% of all nodes. In Fig. 2d, we observed that the majority of OGs in the meta-interactome corresponds to a single protein while the majority of links is composed of one interaction (Fig. 2e, f). Since the average weight of links is 1.0 ± 0.1, we can consider our network as largely unweighted. As a consequence, we found that the average path length in the unweighted network is 3.7 ± 0.9 while the diameter of the network is roughly 15, indicating small world network characteristics [24]. The average number of neighbors is 10.2 ± 23.9, an average that is likely influenced by the presence of several broadly-defined OGs. Since these large OGs contain thousands of members across hundreds of genomes in some cases, we treat them as groups of paralogs [22]. Demonstrating the scale-free tendency of many similar networks [25], we found that the distribution of the number of neighbors has a fat tail.

### Functional annotation of orthologous groups

**Fig. 3:**
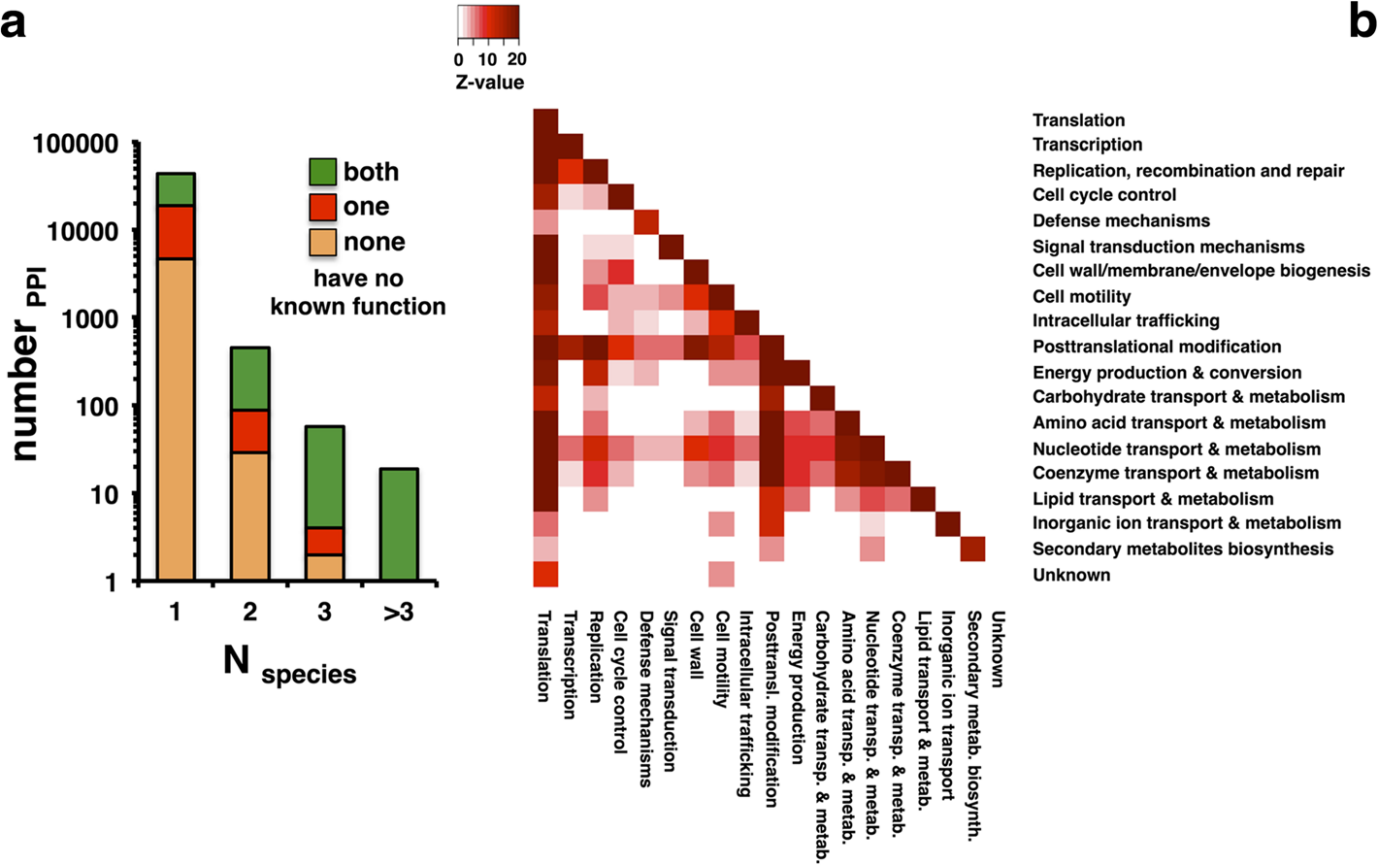
Conserved and cross-functional interactions in the consensus meta-interactome. **a** Counts of PPIs in the consensus meta-interactome network. N_species_ indicates the number of distinct bacterial species contributing the interaction; a value of 1 denotes an interaction observed for a single species only. For each count, subsets denote how many PPIs involve two, one, or zero interactors of known function (as both, one, and none, respectively). **b** Significant connections between functional classes are mediated by the underlying PPIs in the consensus meta-interactome. For each class combination we calculated a Z-score that reflects the significance of the interaction density between classes and class coverage. While interactions mostly appear between the same classes, we also observe that most functional cross-talk emanates from OGs with translational as well as posttranslational functions

Single interactomes are known to have many gaps, indicating PPIs that went undetected in experimental studies [26, 27]. Since a missed interaction in one study may be found in an independent study through evolutionary conservation of the corresponding interacting proteins, a meta-interactome network potentially reveals such gaps. As such, we assume that links between orthologous groups in the consensus meta-interactome may be indicative of undetermined PPIs between orthologs in the corresponding organisms. Counting the number of bacteria a given PPI was observed in we found that relatively few interactions appear in multiple bacterial species (Fig. 3a). In particular, we found 43,116 interactions that occurred only in a single species, 361 appeared in two species while only 68 interactions occurred in three or more species.

Any single bacterial proteome may contain hundreds or even thousands of proteins of unknown or unclear function. Out of more than 43,000 interactions, less than 10,000 involve two interactors of unknown or unclear function (Fig. 3a). Due to limited cross-species overlap, just a small subset of fewer than 100 interactions is observed in more than one species *and* involves one or more interactors of unknown function.

Certain functional groups contribute more extensively to the meta-interactome than others, potentially reflecting the occurrence of more common types of PPIs across bacteria in general. In Fig. 3b, we determined the overrepresentation of functional crosstalk between orthologous groups based on the underlying interactions between different proteins in the consensus meta-interactome. In particular, we determined a log-odds ratio of the observed and expected frequencies of interactions between OGs of the corresponding functional classes, allowing us to calculate a Z-score (see Methods). While most interactions appeared between the same classes, significant cross-talk mostly emanated from OGs with translational as well as posttranslational functions (Fig. 3b).

**Fig. 4:**
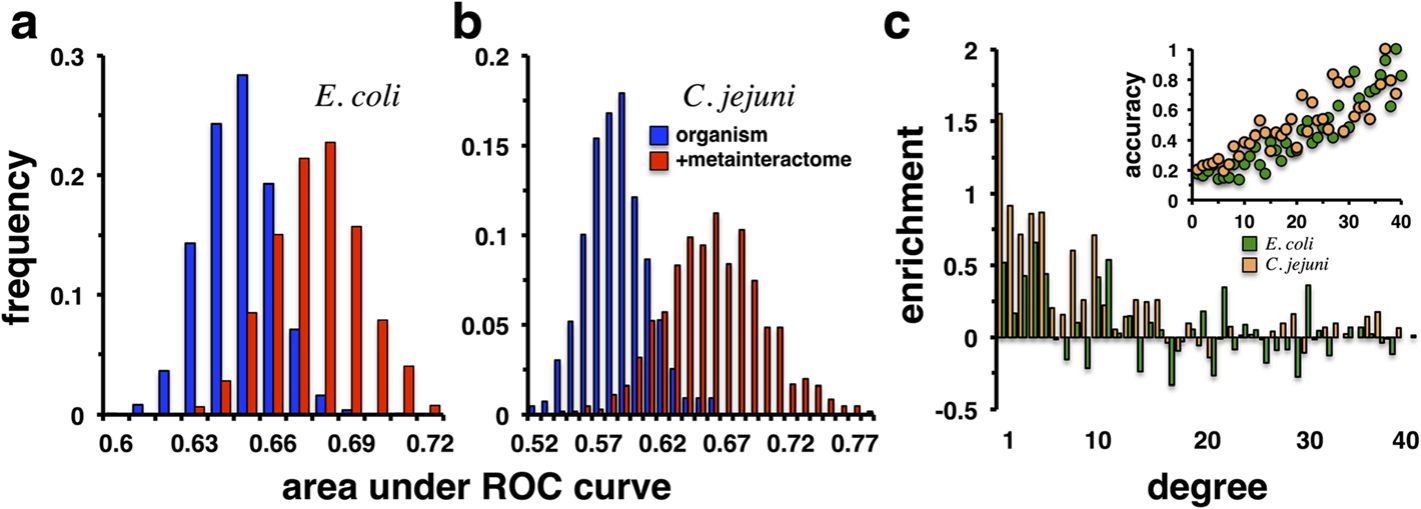
The consensus meta-interactome improves functional predictions. **a** Predicting the functions of sampled OGs we observed that the addition of the consensus meta-interactome allowed for better functional prediction (*P* < 10^-50^, Student’s *t*-test). Connecting functionally annotated orthologous groups (OG) if they harbored interacting proteins of *E. coli* we randomly sampled 20% of all OGs 1000 times and utilized the remainder to predict the functions of the sampled OGs. As a measure of the prediction quality we calculated the area under the ROC curve. In comparison, we augmented the underlying *E. coli* specific network of OGs with remaining links in the underlying consensus meta-interactome. **b** We obtained similar results when we considered OGs that were initially connected by interactions between proteins of *C. jejuni*. In the inset of (**c**) we calculated the fraction of correctly predicted functions of OGs as a function of the degree in the underlying OG networks of *E. coli* and *C. jejuni*, suggesting that increased number of links corresponds to elevated levels of prediction accuracy. Assessing the impact of the consensus meta-interactome on the accuracy of predicted functions of OGs, we observed that the functional prediction for OGs with low degree was improved

To determine the impact of the consensus meta-interactome on our ability to predict functions we generated a network of functionally annotated orthologous groups that were composed of PPIs between proteins in *E. coli*. In particular, we randomly sampled 80% of all functionally annotated OGs 1000 times to predict the functions of the remaining 20%. Using a stochastic model [28] (see Methods) every OG is represented by a profile, reflecting the probability of having a certain function. Applying different probability thresholds for the presence of a functional annotation, we determined ROC curves, and measured the area under the curve as a measure of the prediction quality (Fig. 4a). In comparison, we considered all remaining interactions in the consensus meta-interactome, demanding that each OG was functionally annotated. Analogously, we randomly sampled 20% of annotated OGs that appeared in the original network of OGs based on interactions in *E. coli*. Notably, we observed a shift toward increased values of the area under the ROC curve. Such a difference was statistically significant (*P* < 10^-50^, Student’s *t*-test), suggesting that the augmentation of the underlying network with interactions from other bacteria significantly improved the quality of functional predictions (Fig. 4a). Analogously, we found similar results when we considered PPIs in *C. jejuni* (Fig. 4b, *P* < 10^-50^). Based on our random samples, we calculated the fraction of correctly predicted functions of OGs as a function of the degree in the underlying OG networks of *E. coli* and *C. jejuni* (inset, Fig. 4c). Specifically, we observed that increased number of links corresponds to elevated levels of prediction accuracy of a given OG. In the main plot of Fig. 4c, we assessed the impact of the consensus meta-interactome on the accuracy of predicted functions of OGs. Comparing frequencies of correctly predicted OGs, we found that the prediction of OGs with low degree was especially improved.

**Fig. 5:**
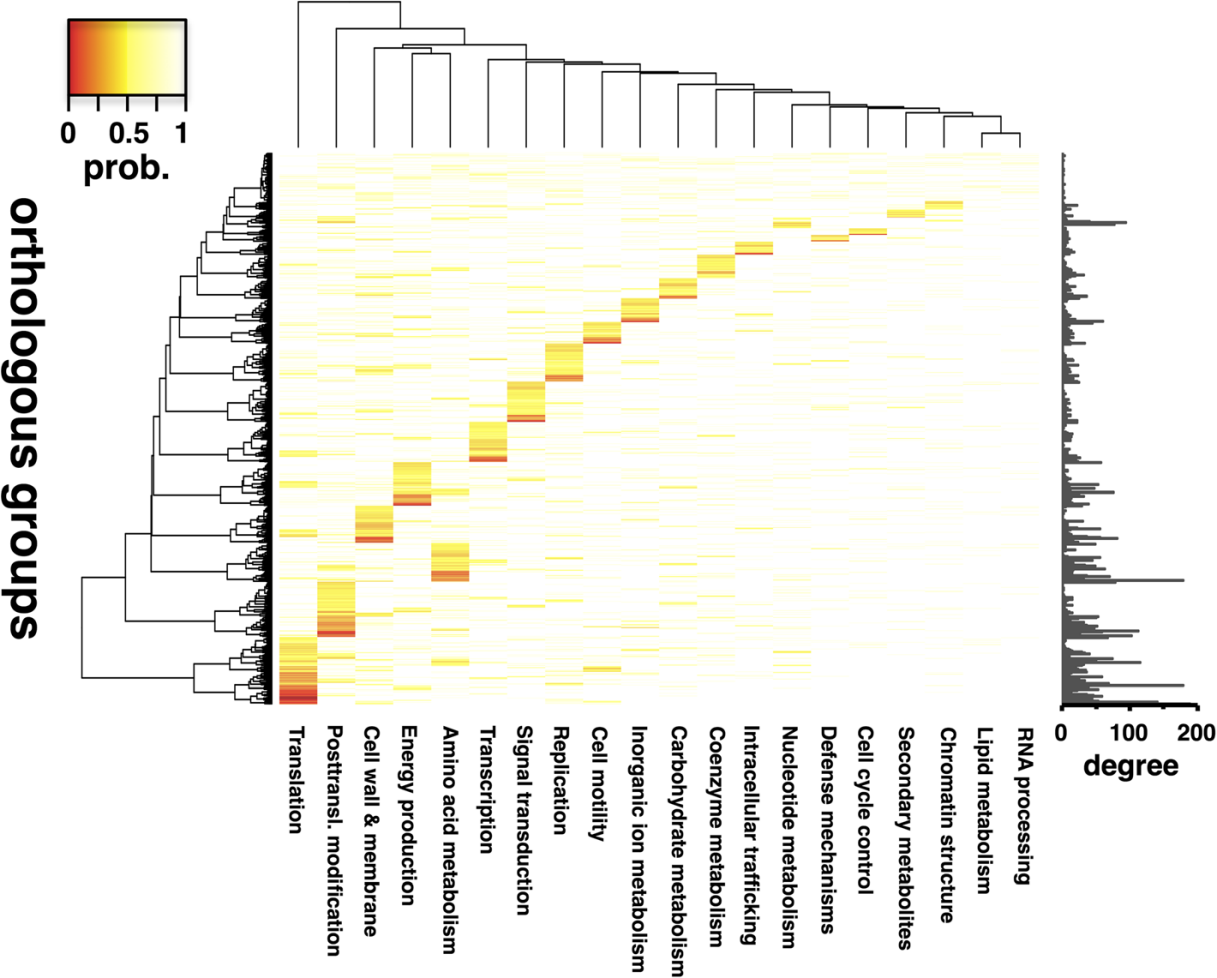
Functional prediction of uncharacterized orthologous groups. Functional similarity of interacting orthologous groups in the network. Each orthologous group (specifically, a node in the network) occupies a single row in the heatmap. A node’s degree in the consensus meta-interactome is shown on the *right*. Each column is a single functional category

To harness the power of the consensus meta-interactome, we used the network of OGs to predict the functions of otherwise functionally unknown orthologous groups. In Fig. 5, we observed that most OGs were clearly involved in translational functions and posttranslational modifications. Notably, such results corresponded well to the observations that most functional crosstalk emanated from these functional classes (Fig. 3b).

### The meta-interactome predicts interactomes and their size

The construction of a meta-interactome as described above can be utilized to predict the interactome of any species with or without interaction data. We used the consensus meta-interactome as a model to predict any potential interactions in a given proteome independently of the availability of protein interactions in the underlying organism. In particular, we considered all interactions between OGs that contain proteins of a given proteome of an underlying organism. As such, we consider all proteins of the given proteome interacting, when we find their corresponding OGs interacting. As a consequence, the interactome of a well-studied species such as *E. coli* can be improved by predicting yet undetected PPIs using data from a related but distinct species.

**Fig. 6:**
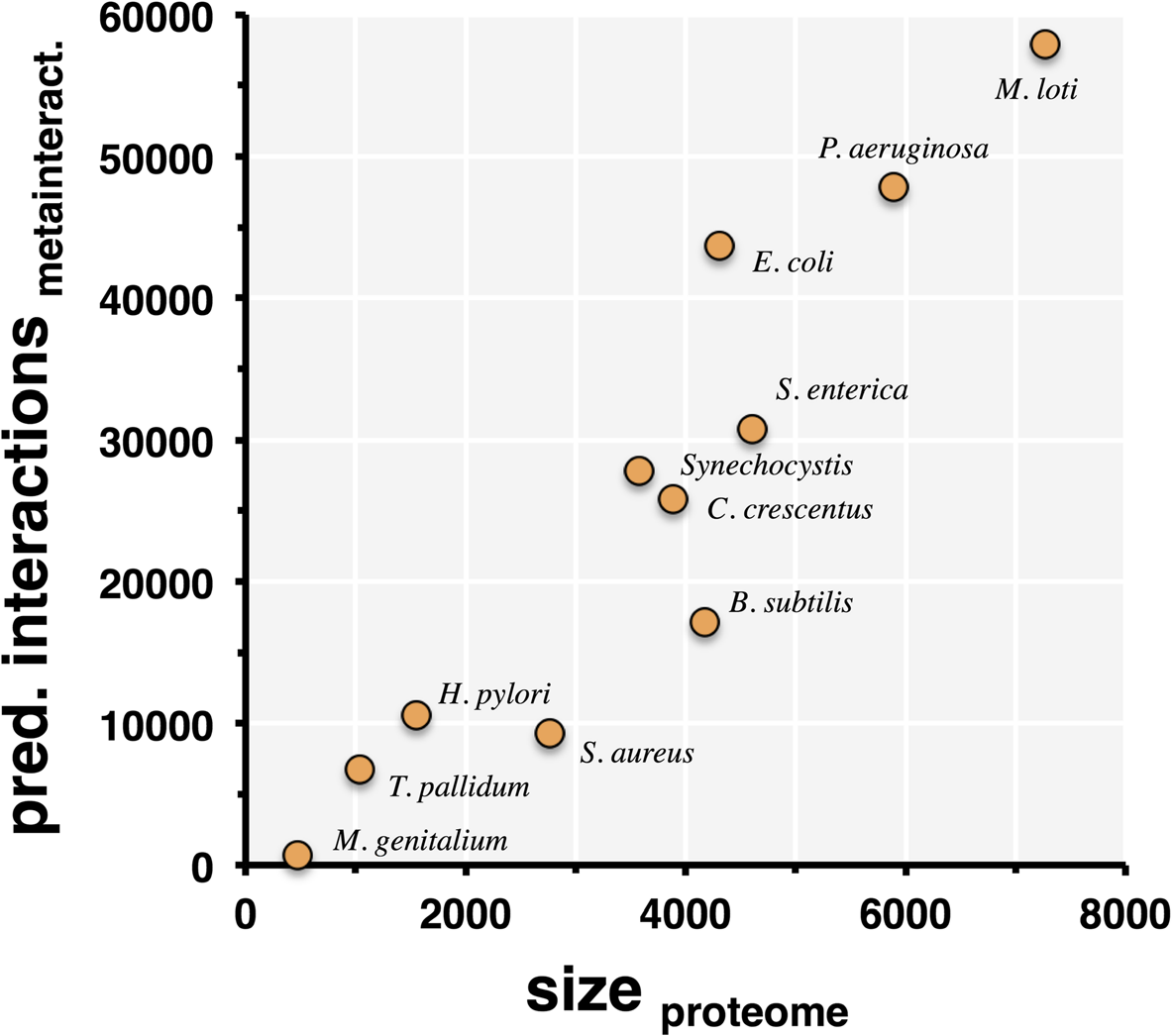
Predictions of maximal interactome size. Based on the consensus meta-interactome we show the upper bounds of predicted interactome size (in number of PPI) as a function of proteome size. Each point corresponds to the Uniprot reference proteome of a single species (see Methods for strain identities and text for details)

This simple prediction method was used with all protein-coding genes from each of several representative bacterial species of varied genome and proteome size (Table 2). Out of all 11 bacterial species shown, six have had comprehensive protein interactomes published, and the data is reflected in the total number of proteins participating in PPIs with experimental evidence. To obtain a starting point for our predictions, we used the interactome size estimation methods developed by Stumpf et al. [7]. These methods primarily depend on the number of interactors and interactions in an experimental interactome to predict the true interactome size and therefore account for interactions not detected in the interactome. Here, we used the Stumpf et al. methods with three different counts of interactors and interactions: those from each of the six published interactomes, the larger counts found in the meta-interactome, and the fraction of the interactome derived from experimental data. In cases where a given species has been the subject of just one comprehensive interactome study (e.g., with *Synechocystis)*, the counts provided by the first option are very similar to the third. Considering a set of reference proteomes (Table 2), we found that interactome sizes thus obtained appear to increase linearly with the proteome size of the underlying bacterial species (Fig. 6). This is based on the assumption that the average number of interactions (or “functions”) per protein remains roughly the same, except in cases of genomes that increased by additional paralogs (which may be involved in additional interactions). For example, the *E. coli* genome codes for more than 4000 unique proteins, and more than 3000 of which have been found to participate in at least one PPI in one or more studies. The *B. subtilis* genome codes for roughly the same number of unique proteins but fewer than 1000 of these proteins have been found to participate in PPIs. However, *B. subtilis* has also been studied much less extensively, hence these numbers do not reflect the true number of interactions in a cell.

**Table 2:** Predicted bacterial interactome sizes

	Predicted interactome^b^ size from …				
Species and Strain Name	Meta-interactome (this study)	Published interactome^a^	Meta-interactome, in PPIs^a^	Meta-interactome (experimental PPI only), in PPIs^a^	Proteins in proteome (vs. proteins in meta-interactome)
*Bacillus subtilis str.* 168	17146	*N/A*	117229	67921	4175 (1597)
*Caulobacter crescentus* CB15	25792	*N/A*	177318	1580788	3885 (1482)
*Escherichia coli* K-12	43702	25736	62770	30087	4306 (3593)
*Helicobacter pylori* 26695	10576	13275	14271	5455	1553 (1337)
*Mesorhizobium loti* MAFF303099	57905	50735	256414	50838	7272 (3456)
*Mycoplasma genitalium* G37	718	*N/A*	7331	*N/A*	475 (149)
*Pseudomonas aeruginosa* PAO1	47815	*N/A*	88143	7073281	5892 (2488)
*Salmonella enterica* subsp. *enterica* serovar Typhi	30788	*N/A*	268219	554147	4607 (2723)
*Staphylococcus aureus* NCTC 8325	9339	*N/A*	59233	650299	2767 (1099)
*Synechocystis* sp. PCC 6803	27816	11221	66575	11811	3575 (2311)
*Treponema pallidum str.* Nichols	6722	7433	10350	7762	1036 (835)

^a^Method of Stumpf et al. [7]

^b^Published interactomes are those specified in Table 1

The interactome size prediction methods in this study are the results of predicting that two different orthologous group members will interact as long as members of the two groups have been observed interacting in any bacterial species. The resulting totals are shown in the *second column* (Predicted interactome size from meta-interactome (this study)). Results from the interactome size prediction method used by Stumpf et al. [7] are shown here for comparison: where possible, these are used with interaction and interactor totals from published interactomes (*third column*). Two hybrid approaches are also presented, with the input for the Stumpf method provided by the total counts of interactors and interactions predicted by the interactome (*fourth column*) or by the experimentally-observed interactions in the meta-interactome only (*fifth column*). The final column provides the count of proteins in each respective proteome along with the fraction of those proteins present in the meta-interactome, including all proteins involved in functional predictions

The more interactions are detected, the fewer are left to be predicted. As a result, unstudied or incomplete interactomes have the largest potential for prediction. For instance, there are very few PPIs known from *Streptococcus pneumoniae*: just 63 of the 2030 proteins coded for in the *S. pneumoniae* R6 genome have experimental interactions in IntAct. Our predicted interactome for this protein set increases that total to 850 proteins (Fig. 6). Similar results are seen for *B. subtilis* and for *Mycobacterium tuberculosis*.

## Discussion

### Biological differences vs. technical differences in interactomes

Published interactomes vary in size and composition across different studies and species, rendering them difficult to compare. In the case of *Campylobacter jejuni*, a genome of 1654 ORFs yielded an interactome of more than 11,000 distinct PPIs from yeast two hybrid (Y2H) screens using ~90% of the ORFs, or 1477 in total [29]. By contrast, the interactome of *M. loti* as reported by Shimoda et al. [30] includes just over 3100 PPI though its proteome contains 7281 predicted proteins. These discrepancies are clearly determined by different coverage: in the case of the *M. loti* interactome, the full genome was used as yeast two hybrid preys but only 1542 of 7281 genes were used as baits. This subset was curated *as per* the goals of the study and therefore represents a conscious technical difference between interactomes.

The comparison of interactomes also reveals unavoidable methodological discrepancies. More than half of the PPIs contributing to the meta-interactome were observed using two hybrid methods, offering some methodological consistency, yet these methods may vary in technical implementation details such as protein expression conditions, growth conditions, or even the exact yeast or bacterial strains used. As we have shown previously, even when exactly the same protein pairs are tested by Y2H assays, small differences in the experimental protocol can yield dramatically different results [31]. Inclusion of affinity purification and mass spectrometry (AP/MS) approaches introduces another concern: AP/MS methods typically *infer* interactions from co-purification through a spoke model approach (that is, that a single bait is assumed to interact with all of its co-purified proteins) while two hybrid methods generally screen for binary interactions only. We have previously estimated that the spoke-model approach over-estimates the number of PPIs by about 3-fold [8].

In this study, we have attempted to reduce the impact of technical differences between interaction studies by focusing on the subset of interactions that we pooled from multiple species. This approach is especially effective for minimizing the influence of potentially erroneous spoke model interactions, as the bulk of these interactions in the meta-interactome are from just two species (*E. coli* and *M. pneumoniae,* both of which have been subjects of full protein complex surveys). In the meantime, we believe a cross-species approach is helpful for identifying expected PPI in interactomes. As seen in Fig. 3a, fewer than one thousand OG vs. OG interactions in the meta-interactome have been observed in more than one bacterial species, yet more interactions should be conserved across any two pairs of bacterial species.

Finally, some differences among interactomes may be due to real distinctions in genetics and physiology. Many processes show considerable genetic variation in bacteria, even when they are traditionally considered to be highly conserved. For instance, ribosomes are surprisingly malleable [32, 33] as are flagella [17], cell division proteins [34] or protein complexes in general [35]. A more complete meta-interactome should therefore shed light on the biological differences between species.

### Meta-interactomes reveal broadly-conserved interactions involving proteins of unknown function

Of all OG-OG interactions involving OGs of unknown or unclear function (UF OGs), fewer than 10 are seen in more than 2 different species (Fig. 3a). Highly conserved PPIs are thought to serve more fundamental processes in a cell (e.g. [8, 12]), hence we identified well-conserved interactions for function prediction. The most frequently observed PPIs (specifically, OG-OG interactions) across species are interactions among enzyme subunits, e.g. the alpha and beta subunits of tryptophan synthase which is a well-studied interaction. A selection of interactions involving interactors of less clear function are shown in Table 3.

**Table 3:** Conserved interactions involving OGs of unclear function

Interactor A (ENOG…) -- Functional Category	Interactor B (ENOG…) -- Functional Category	Function (A)	Function (B)	Species
4105W16 - S	4105W16 - S	Blue light sensor protein	Blue light sensor protein	Synechocystis sp. PCC 6803, *Thermosynechococcus elongatus*
4105CXV - S	4108XPN - S	Gliding motility protein	Roadblock lc7 family protein	*Thermus thermophilus, Myxococcus xanthus*
4108WXF - S	4108WXF - S	KaiA, Component of the KaiABC clock protein complex	KaiA, Component of the KaiABC clock protein complex	*Thermosynechococcus elongatus, Synechococcus elongatus*
4105K7D - S	4108UKE - J	Ribosome maturation factor RimP	30S ribosomal protein S12	*Campylobacter jejuni, Helicobacter pylori*
4105ZRE - S	4108YZA - E	Protein of unknown function (DUF3539)	GlnB, Nitrogen regulatory protein P-II	*Nostoc sp.* PCC 7120*, Synechococcus elongatus*
4105QDU - S	4108V9G - S	Uncharacterized protein	Uncharacterized protein	*Campylobacter jejuni, Helicobacter pylori*
4108SDW - S	4107QMP - L	Recombination protein RecO	DNA polymerase III gamma and tau subunits	*Campylobacter jejuni, Helicobacter pylori*

All interactions in this table have been observed in at least 4 PPIs across bacterial species of at least two different genera, with species identified in the Species column. A more complete list and an explanation of abbreviations can be found in Additional file 5

This list omits broadly-conserved self-interactions, such as those among histidine kinases (ENOG4105BZU). An orthology-based approach is more informative when used with interactions among proteins in different groups (in this case, different OGs) than with interactions among proteins of the same OG as individual protein identities are ignored in the consensus meta-interactome. We have made the assumption that cross-OG interactions are more likely to indicate cross-function interactions and are therefore of great relevance to functional context. This idea is illustrated by the MdB and NDH-1 complexes:

### MdaB

(ENOG4105NF4) proteins figure prominently in the meta-interactome. MdaB was first identified as modulator of drug activity [36] and is still annotated as such in most databases. Later, Wang et al. (2004) characterized it as a novel antioxidant protein similar to NADPH nitroreductases which play an important role in managing oxidative stress essential for successful colonization of *H. pylori* in its host [37]. Its mutants are unable to colonize human host cells [37]. However, the MdaB interaction network indicates another unrelated function as it interacts with three motility related proteins in three different species: a chemotaxis protein (UniprotKB: O25152) from *H. pylori*, flagellin C (UniprotKB: P96747) from *C. jejuni*, and chemotaxis protein CheW (UniprotKB: P0A964) from *E. coli* K-12. We suggest that the colonization phenotype is related to its motility rather than oxidative stress. In fact, motility is critical for initial colonization of *H. pylori* in its host cells [38]. FlaC in particular is well characterized as an important factor for host cell invasion in *C. jejuni* [39].

### NDH-1 complexes

**Fig. 7:**
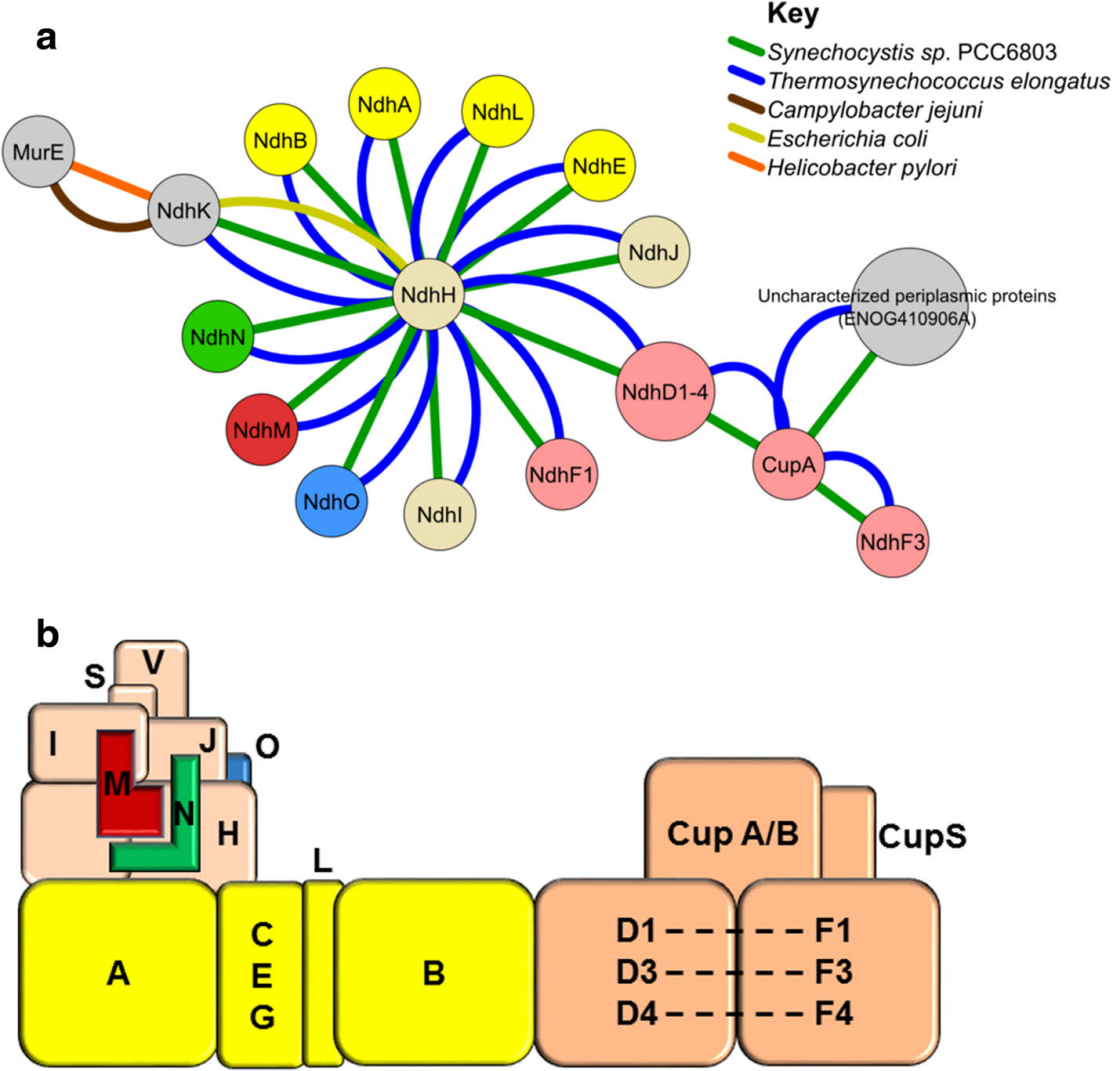
The NDH-1 complex as an example of conserved interactions. **a** An NDH component interaction network from multiple species. Each node in this network corresponds to a single orthologous group and is labeled with the corresponding group member in cyanobacteria (i.e., the sources of most of the PPI observed for NDH complex members). Groups are colored as in Part B; groups in *gray* have predicted accessory functions. Interactions between any proteins in two groups are shown as edges. Edges are colored as noted in the Key. **b** A model of the NDH-1MS complex in cyanobacteria. Figure after [54]. Each box corresponds to a protein or group of proteins; those labeled with a single letter are Ndh proteins. *Dotted lines* indicate alternate complex forms. See [54] for further details

Interactions between components of a protein complex can be reconstructed from the meta-interactome interactions. The cyanobacterial NDH-1 membrane protein complexes provide a good example: these proteins belong to widely-conserved family of energy converting NAD(P)H: Quinone oxidoreductases which are unique to organisms capable of photosynthesis. Many distinct NDH-1 complexes may coexist in cyanobacteria to carry out different functions like respiration, cyclic electron transfer and CO_2_ uptake [40–42]. At least four NDH-1 complexes are predicted in cyanobacteria in *Synechocystis* 6803, usually called L, L’, MS, and MS’. Each complex is composed of a basal complex (NdhA-C, NdhE,G-K, NdhL-O) associated with variable subcomplexes of Ndh and Cup subunits (Fig. 7). Each complex has a different function: for example, NDH-1 L and L’ are responsible for respiration and cyclic electron flow and NDH-1MS/MS’ for CO_2_ uptake. The multitude of functionality of cyanobacteria is possible due to the presence of a great diversity of ndhD (D1-D6) and ndhF (F1, F3 and F4) gene families. It is possible that with sudden changes in CO_2_ levels, cyanobacteria can flexibly use the NDH-1 M basal subcomplex and change contents of its variable subcomplex to form MS and L complexes [42].

An example of the NDH-1MS (NDH-1 M, NdhD/F/CupA/CupS) network in *Synechocystis* 6803 and *T. elongatus* BP-1 is shown in Fig. 7a and a corresponding model is provided in Fig. 7b. Only one similar interaction (NuoD and NuoB) is observed in *E. coli*. CupA (ENOG4107YAI) has been found to interact with NdhF (ENOG4106TXZ), NdhD1-D4 (ENOG4105C8S), and an unknown protein (ENOG410906A) to form the NDH-IS (NdhD/F/CupA/CupS) sub-complex. Members of ENOG410906A, though coding for protein of unknown function, have sequence similarity to Fasciclin superfamily proteins associated with cell adhesion in plants and algae species. Korste et al. (2015) [42] found a similar protein (UniprotKB: P73392) in *Synechocystis* 6803 and Q8DMA1 in *T. elongatus* BP-1 and designated it as CupS, a small subunit of the NDH-1MS complex [42]. The NMR studies showed that though the protein was structurally similar to the Fasciclin superfamily, but was not associated with adhesion, contrary to Fasciclin superfamily proteins, given its intracellular location. Though CupS has been shown to interact with NdhD/NdhF/CupA, its function is still unknown. This network data not only provides clarity about the interaction of NDH-1 complex proteins but also predicts a probable respiratory function for the members of ENOG410906A. Cyanobacterial meta-interactome networks (Fig. 7a) clearly show that the NdhH subunit interacts directly with all associated subunits, a point which had been missing in all predicted structures of NDH-I.

### Meta-interactomes predict interactome sizes

If we assume that the average degree of a protein remains the same, independent of the proteome, then interactomes should grow linearly with proteome size and thus with genome size (Fig. 6). However, bias in the available data is likely creating distorted predictions: the *E. coli* data point (at the top of the figure) nearly does not fit the trend and most of the PPI predictions we can make originate with *E. coli* data. Additionally, predicted interactome sizes are limited by the number of unannotated or highly unusual genes in a genome. The largest genomes in this set, from *P. aeruginosa* and *M. loti,* contain ~310 and 737 genes without orthology predictions, respectively. Further annotation of these genes or interactions among their products may allow for interaction predictions more like those for other species. Total counts of OG vs. OG interactions corresponding to each taxon in the meta-interactome are provided in Additional file 6; in many cases, the meta-interactome contains just one interaction for a given species and hence suggests candidates for further exploration (here, taxon IDs are used instead of species to avoid counting interactions multiple times for closely related species or strains).

Proteome size is likely just one trait contributing to the overall complexity of a species [43] and we may consider the interactome of that species to represent one facet of its complexity. Some methods used to estimate interactome size were intended for use with human or yeast proteins rather than those from bacteria [44, 45]. Another confounding factor is that false positives are likely to grow exponentially with increasing proteome size, e.g. because a fraction of proteins interact non-specifically with hydrophobic surfaces.

The meta-interactome approach is an intentional abstraction. It is intended to underscore the bacterial cross-species commonality and conservation of protein interactions among currently available interaction data. As a result, this approach is limited by at least three main factors: limitations of protein-protein interaction screens, limitations of publicly-available data, and constraints on orthology prediction. All experimental interactomes are inherently incomplete and may include numerous false positives and otherwise erroneous results. The authors of these studies employ different filtering approaches and likely interpret their results based on expectations (e.g., some interactome studies eliminate frequently-interacting proteins like chaperones from their screens). Most of the available interaction data for bacterial proteins has focused on just a handful of species. Additional screens of proteins from more diverse sources across the bacterial tree of life will reveal a universe of yet unknown functions, just as gene sequences did for genetic diversity.

## Conclusions

In this work, we have assembled a set of more than 52,000 unique PPIs between bacterial proteins to perform cross-species interactome comparisons. The combined set, or meta-interactome, allows us to define a set of interactions observed across multiple species. Though this set is much smaller than expected, this result highlights the ongoing challenge of duplicating results of interactome screens. In an effort to address this challenge, we use the meta-interactome as a model for bacterial species without comprehensive interactome results, such as *Bacillus subtilis* and *Salmonella enterica.* We also employ the meta-interactome as a predictive tool to assign functions to uncharacterized proteins. These efforts and the methods presented here will allow researchers pursuing new interactome studies to easily predict the potential scope of their own results. As more bacterial interactomes reach completion, the interactions occupying prominent locations in a meta-interactome will likely reveal novel, broadly-conserved biological phenomena and appealing anti-microbial targets.

## Methods

### Literature mining for citation analysis

The initial stages of this project required assessment of whether comparisons of bacterial interactomes were common in the interactome literature. A list of 11 publications, each describing a single bacterial protein-protein interactome, was assembled as a representative set of the bacterial protein-protein interactome literature, namely those of *H. pylori* [12, 46], *C. jejuni* [29], *Synechocystis* [47], *M. loti* [23], *T. pallidum* [17], *E. coli* [8, 9], *M. pneumoniae* [48], *M. tuberculosis* [21], and *S. aureus* [49]. The full list of citations from each paper was retrieved from PubMed Central in XML format in August 2015. All citation lists were combined to determine citations shared by multiple publications in the set. Publications citing multiple representative interactome publications are those with potential for cross-interactome comparisons (see Additional file 2 for the list of publications in the set and their corresponding citations).

### PPI data sets

The full set of interactions was obtained from the IntAct database [3]. To produce the data set used in this study, the full set of IntAct interactions was filtered by Uniprot taxonomy to include only protein-protein interactions (PPI) from bacterial sources (species:"taxid:2"). Prior to further filtering, this interaction set includes 63,421 PPI across all interaction types. All interactions without Uniprot identifiers (i.e., interactions involving ChEBI chemicals) were removed, as were interactions with erroneous annotation (i.e., interactions involving bacterial proteins vs. eukaryote proteins). The set of IntAct interactions was augmented with the protein interactome of *Mesorhizobium loti* [23]. Where possible, proteins were assigned membership in orthologous groups (OGs) using eggNOG v.4 NOGs [19]; proteins without OG annotation are treated as single-member OGs and referred to using their UniprotAC identifiers. All PPIs are retained in the data set regardless of experimental observation method; interactions derived from spoke-expansion models are treated identically to those defined as “direct” interactions.

### Construction of meta-interactome networks

PPI sets were obtained and filtered using a set of scripts developed for the purpose, *Network_umbra* (available at https://github.com/caufieldjh/network-umbra). This program parses interaction data files in PSI-MI TAB 2.7 format (MITAB27; a format used by protein-protein interaction databases; developed by the HUPO Proteomics Standards Initiative and described in detail at https://code.google.com/p/psimi/wiki/PsimiTab27Format) and facilitates all further methods described in this study.

The full set of PPIs sourced from IntAct constitutes the starting data set for meta-interactome construction [3]. We define a meta-interactome as a set of PPIs where similar proteins and the interactions among those proteins are merged into single interactor groups and interactions. Interactions among proteins of the same group are considered a self-interaction, though all interactions retain properties of the source interaction network, including the count of PPIs and count of unique source species contributing to the interaction. Meta-interactome groups are defined by eggNOG v.4 NOGs [19]. Because annotations for interactions involving similar proteins from closely-related species may differ, the species and strains corresponding to each interaction were labeled using NCBI Taxonomy identifiers and identifiers sharing a parent or a child were merged. All interactions were compressed using OG-annotated proteins such that each OG-OG interaction appears in each data set only once per species, though a protein may belong to multiple OGs (in these cases, the resulting OG name includes both identifiers separated by a comma, e.g. "COG1100,COG4886").

The full meta-interactome is provided in Additional file 3 in PSI-MI TAB 2.7 format, with the addition of orthologous groups in the final two columns (corresponding to interactors A and B, respectively). This interactome contains 52,734 interactions among 12,706 unique proteins, 1805 (3.4%) of which fail to map to an orthologous group. Treated as a network of OGs, this network contains 8521 unique interactors.

A further subset of the meta-interactome was prepared such that this set merged all interactions on the basis of shared interactors (see Additional file 4). For example, two different interactions between proteins in OG1 and proteins in OG2 are considered a single interaction. Furthermore, each OG-OG interaction is counted as a single interaction across any number of species. We refer to this set as the consensus meta-interactome. This network contains 8475 unique interactors and 43,545 interactions.

### Interactome size prediction

We utilized the consensus meta-interactome of OG-OG interactions to generate predicted interactomes for a given bacterial species. Given a list of UniprotAC identifiers we assigned each to an OG and constructed a set of interactions among those OGs based on their presence in the consensus network. In most cases, predictions are general and unverified: if a pair of OGs is present in the consensus network they are predicted to interact in any context. Reference proteomes for the following species and strains were used, with NCBI taxonomy IDs in parentheses: *Bacillus subtilis* str. 168 (224308)*, Caulobacter crescentus* CB15 (190650), *Escherichia coli* K-12 (83333)*, Helicobacter pylori* 26695 (85962)*, Mesorhizobium loti* MAFF303099 (266835), *Mycoplasma genitalium* G37 (243273), *Pseudomonas aeruginosa* PAO1 (208964), *Salmonella enterica* subsp. *enterica* serovar Typhi (90370), *Staphylococcus aureus* subsp. *aureus* NCTC 8325 (93061), *Synechocystis* sp. PCC 6803 substr. Kazusa (1111708), and *Treponema pallidum* subsp. *pallidum* str. Nichols (243276).

### Functional prediction of unknown proteins in *S. pneumoniae*

We modeled the prediction of a functional class *σ* of a protein *i* as a Potts model [28]. In particular, we considered functional annotation of proteins in *S. pneumoniae* using COG classes. All proteins without a functional annotation as well as proteins that were either classified as ‘unknown’ or had a ‘general function’ were randomly assigned a function out of the remaining 23 classes. In particular, we minimized the following global function, $E=-{\displaystyle \sum_{i, j}{J}_{i j}\delta \left({\sigma}_i,{\sigma}_j\right)}-{\displaystyle \sum_i{h}_i\left({\sigma}_i\right)}$

where *J*_ij_ is the adjacency matrix of the interaction network for the unclassified proteins. In particular, *J*_ij_ = 1 if unclassified proteins *i* and *j* interact and vice versa. (*i*,*j*) is the discrete $\delta$ function, and *h*_i_(*σ*_i_) is the number of classified interaction partners of protein *i* with function *σ*_i_.

To minimize *E* we applied a simulated annealing approach that features an effective temperature *T*. After initially assigning random functions to all unclassified proteins, we randomly selected a protein, changed its function to a different class and determined the energy of the new configuration. If the difference of energies ΔE ≤ 0, the new configuration was accepted. If ΔE > 0, the new configuration was accepted with probability *p* = *e* ^− *ΔE*/*T*^. To obtain stabilized functional configurations we repeated such a Monte-Carlo step 10,000 times. Subsequently, we increased the inverse of T by 0.01 in each step and repeated such Monte-Carlo steps. Since minimum energy solutions are not unique, we repeated such runs of simulated annealing 100 times, and considered the fraction of times an unclassified protein *i* was observed in a certain functional state *σ* as an estimate of the probability that protein *i* belongs to class *σ*.

### Interactions between functional classes

Focusing on a set of PPIs that connect proteins in orthologous groups (OG), we counted the occurrence of different class combinations. For each combination of classes *i, j* we determined its probability,

$$ {p}_o\left( i, j\right)=\frac{n_{ij}}{N}, $$

where *N* is the total number of interactions between classes. As a null-model, we determined an expected probability of interactions between classes *i*, *j* ${p}_e\left( i, j\right)=\frac{\left({v}_i{v}_j\right)-\frac{J_{i j}^2}{2}}{\frac{N\left( N-1\right)}{2}}$.

Specifically, *v*_*i*_ is the number of viable proteins in class *i* (i.e. proteins of class *i* that are involved in at least one interaction in the underlying set), and *J*_*i,j*_ is the number of genes that are involved in both classes. Combining these probabilities, we determined a log-odds ratio,

$$ r=\frac{p_o{\left(1-{p}_o\right)}^{-1}}{p_e{\left(1-{p}_e\right)}^{-1}}. $$

For large samples, we estimated the variance of the odds distribution as *σ* ^2^ = *n*_*ij*_^− 1^ + (*N* − *n*_*ij*_)^− 1^ + *a* ^− 1^ + (*b* − *a*)^− 1^

where

$$ \begin{array}{l}\begin{array}{cc}\hfill a=\left({v}_i{v}_j\right)-\frac{J_{i j}^2}{2}\hfill & \hfill \mathrm{and}\hfill \end{array}\\ {} b=\frac{N\left( N-1\right)}{2}.\end{array} $$

In particular, we calculated a Z-score representing the significance of a link between two classes by [18]

$$ Z=\frac{r}{\sigma} $$

### Enrichment of accuracy as a function of degree

To compare the prediction results we obtained with the original networks that were based on interactions in *E. coli* and *C. jejuni* and the complete network of orthologous groups (OG) we calculated the fraction of correctly predicted functions in bins of OGs with a given number of interaction partners in the underlying networks obtained with the mentioned bacterial species. Since each OG was assigned to a functional class with a certain probability, we labeled each group with the most probable function. We defined the enrichment of accuracy in a given bin of degree *k* as

$$ {E}_k= l{g}_2\left(\frac{f_{k, m}}{f_k}\right), $$

where *f*_*k*_ is the fraction of correctly predicted functions of OGs with degree *k* in the original networks. In turn, *f*_*k,m*_ reflects the rate of correctly predicted functions using the consensus meta-interactome.

## Additional files

Additional file 1: Guide to content provided in the supporting tables. (DOCX 20 kb)
Additional file 2: Review of literature citing multiple bacterial interactomes. (XLS 359 kb)
Additional file 3: All interactions in the meta-interactome network. Interactions are provided in PSI-MI TAB 2.7 format, with the addition of orthologous group identifiers for interactor A and B in the 43rd and 44th columns (AQ, AR), respectively. (XLS 47313 kb)
Additional file 4: All interactions in the consensus meta-interactome network. (XLS 6135 kb)
Additional file 5: Conserved interactors of unclear function. (XLS 29 kb)
Additional file 6: Contributions of individual bacterial taxons to the consensus meta-interactome. (XLS 42 kb)

## Abbreviations

AP/MS: Affinity purification and Mass spectrometry
OG: Orthologous group
PPI: Protein-protein interaction
Y2H: Yeast two hybrid
