# Supplementary material for: Bacterial protein meta-interactomes predict cross-species interactions and protein function
Source: BMC Bioinformatics. 2017 Mar 16;18:171. doi: 10.1186/s12859-017-1585-0 (PMC5353844; doi:10.1186/s12859-017-1585-0)
Supplement: Additional file 1: — Guide to content provided in the supporting tables. (DOCX 20 kb) [file 12859_2017_1585_MOESM1_ESM.docx]

**Additional file 1 - Text S1**

**Supplementary Information for**

**"Bacterial Protein Meta-Interactomes Predict Cross-Species Interactions and Protein Function"**

See Materials and Methods for further detail regarding data sources.

In Additional Files 3 and 4 and in the manuscript, counts of orthologous groups are treated in two distinct ways.

All orthology assignments are applied using orthologous groups (OGs) from eggNOG v.4 (Powell et al., 2014; http://eggnogdb.embl.de/).

All proteins were mapped to eggNOG NOG identifiers where possible, preferentially using bacteria-specific NOGs (bactNOGs), followed by more broadly-defined NOGs (root level NOGs, sometimes identified as COGs). See Powell et al. (2014) for descriptions of these orthologous group types. Proteins not mapping to OGs of any type are treated as single-member OGs.

OG counts are those in which each locus has been mapped to an OG and multiple OG members are combined. When more than one locus maps to the same OG, all are treated as a single OG. Loci mapping to multiple OGs are mapped to a new OG with both identifiers, e.g. "COG1100,COG4886").

**Additional file 2**

**S1** Review of literature citing multiple bacterial interactomes.

All publications in PubMed Central were searched for citations for at least one of the 11 pulblications listed below, each of which describes a comprehensive bacterial protein-protein interactome.

PMCID Pubmed Central identifier of the publication.

DOI DOI of the publication.

Publication Title Title of the publication.

Citations The total count of papers, of those listed below, cited by the specified publication.

Rain 2001 Citation to Rain JC, Selig L, De Reuse H, Battaglia V, Reverdy C, et al. (2001) The protein-protein interaction map of *Helicobacter pylori*. Nature 409: 211–215.

Parrish 2007 Citation to Parrish JR, Yu J, Liu G, Hines J a, Chan JE, et al. (2007) A proteome-wide protein interaction map for *Campylobacter jejuni*. Genome Biol 8: R130.

Sato 2007 Citation to Sato S, Shimoda Y, Muraki A, Kohara M, Nakamura Y, et al. (2007) A large-scale protein-protein interaction analysis in synechocystis sp. PCC6803. DNA Res 14: 207–216.

Shimoda 2008 Citation to Shimoda Y, Shinpo S, Kohara M, Nakamura Y, Tabata S, et al. (2008) A large scale analysis of protein-protein interactions in the nitrogen-fixing bacterium *Mesorhizobium loti*. DNA Res 15: 3–11.

Titz 2008 Citation to Titz B, Rajagopala S V., Goll J, Häuser R, McKevitt MT, et al. (2008) The binary protein interactome of *Treponema pallidum* - The syphilis spirochete. PLoS One 3: e2292.

Hu 2009 Citation to Hu P, Janga SC, Babu M, Díaz- Mejía JJ, Butland G, et al. (2009) Global functional atlas of *Escherichia coli* encompassing previously uncharacterized proteins. PLoS Biol 7: 0929–0947.

Kuhner 2009 Citation to Kühner S, van Noort V, Betts MJ, Leo-Macias A, Batisse C, et al. (2009) Proteome organization in a genome- reduced bacterium. Science 326: 1235– 1240.

Wang 2010 Citation to Wang Y, Cui T, Zhang C, Yang M, Huang Y, et al. (2010) Global protein- protein interaction network in the human pathogen *Mycobacterium tuberculosis* H37Rv. J Proteome Res 9: 6665–6677.

Cherkasov 2011 Citation to Cherkasov A, Hsing M, Zoraghi R, Foster LJ, See RH, et al. (2011) Mapping the Protein Interaction Network in Methicillin-Resistant *Staphylococcus aureus*. J Proteome Res 10: 1139–1150.

Hauser 2014 Citation to Häuser R, Ceol A, Rajagopala S V, Mosca R, Siszler G, et al. (2014) A second-generation protein-protein interaction network of *Helicobacter pylori*. Mol Cell Proteomics 13: 1318–1329.

Rajagopala 2014 Citation to Rajagopala S V., Sikorski P, Kumar A, Mosca R, Vlasblom J, et al. (2014) The binary protein-protein interaction landscape of *Escherichia coli*. Nat Biotechnol 32: 285–290.

**Additional file 3**

**S2** All interactions in the meta-interactome network.

*Interactions are provided in PSI-MI TAB 2.7 format, with the addition of orthologous group identifiers for interactor A and B in the 43rd and 44th columns, respectively.*

**Additional file 4**

**S3** All interactions in the consensus meta-interactome network.

InteractorA The first interactor. Either an eggNOG OG identifier or a Uniprot protein identifier, representative of a single-member OG.

InteractorB The second interactor. Either an eggNOG OG identifier or a Uniprot protein identifier, representative of a single-member OG.

*For InteractorA and InteractorB, interactors mapping to multiple OGs include all corresponding OGs, separated by commas. For purposes of this data set, multiple-OG interactors are treated as unique OGs, even if their mappings overlap with other OGs.*

InteractionCount Count of individual PROTEIN interactions contributing to this consensus interaction, as per the meta-interactome.

TaxonCount Count of different taxons (here, a proxy for species) corresponding to the interaction.

*Similar taxons have been grouped together where possible, e.g. two different E. coli K-12 strains are just considered E. coli K-12.*

Taxons The taxons corresponding to this interaction.

FuncCatA Functional category of the first interactor.

DescA Description of the first interactor.

FuncCatB Functional category of the second interactor.

DescB Description of the second interactor

*For all FuncCats and Descriptions, multiple-OG interactors include all annotations, separated by pipe* ( *|* ) *symbols. NA indicates that a functional category or description is not available.*

**Additional file 5**

**S4** Conserved interactions of unclear function.

*The format of this table is identical to that of S3 Table (Additional file 4).*

*All interactions in this table are those observed in at least two distinct species and involving at least one interacting OG with a functional category annotation of* “S”.

**Additional file 6**

**S5** Contributions of individual bacterial taxons to the consensus meta-interactome.

*This table lists individual bacterial taxons and NCBI taxonomy IDs by their contributions to the consensus meta-interactome, in total numbers of OG vs. OG interactions from any source. Taxons are used in order to avoid counting interactions from closely related species or strains more than once, e.g. all strains of* E. coli *K-12 are considered in the same context and are collectively referred to as* Escherichia coli *or* *taxonomy ID 83333.*
